# Crowdsourcing-Assisted Radio Environment Database for V2V Communication †

**DOI:** 10.3390/s18041183

**Published:** 2018-04-12

**Authors:** Keita Katagiri, Koya Sato, Takeo Fujii

**Affiliations:** 1 Advanced Wireless and Communication Research Center (AWCC), The University of Electro-Communications, 1-5-1 Chofugaoka, Chofu-shi, Tokyo 182–8585, Japan; fujii@awcc.uec.ac.jp; 2 Department of Electrical Engineering, Faculty of Engineering, Tokyo University of Science, 6-3-1 Niijuku, Katsushika-ku, Tokyo 125-8585, Japan; k_sato@ieee.org

**Keywords:** wireless distributed networks, spectrum database, radio propagation

## Abstract

In order to realize reliable Vehicle-to-Vehicle (V2V) communication systems for autonomous driving, the recognition of radio propagation becomes an important technology. However, in the current wireless distributed network systems, it is difficult to accurately estimate the radio propagation characteristics because of the locality of the radio propagation caused by surrounding buildings and geographical features. In this paper, we propose a measurement-based radio environment database for improving the accuracy of the radio environment estimation in the V2V communication systems. The database first gathers measurement datasets of the received signal strength indicator (RSSI) related to the transmission/reception locations from V2V systems. By using the datasets, the average received power maps linked with transmitter and receiver locations are generated. We have performed measurement campaigns of V2V communications in the real environment to observe RSSI for the database construction. Our results show that the proposed method has higher accuracy of the radio propagation estimation than the conventional path loss model-based estimation.

## 1. Introduction

Along with the rapid development of wireless communication technology, the number of mobile terminals has significantly increased during the last decade. As can be seen from a prediction by Cisco [1], 50 billion terminals will be connected to the Internet by 2020, and an enormous number of wireless terminals will communicate in various environments under the finite spectrum resources. In order to efficiently utilize the limited spectrum, it is important to appropriately select communication parameters such as frequency and transmission power according to the radio environment characteristics between terminals. Therefore, a radio propagation estimation becomes a crucial issue in the future wireless communication systems.

The use of the empirical propagation model is the most fundamental method for the radio propagation estimation because these models had been constructed by many discussions with the huge measurement campaigns in various environments. Although the accuracy of the path loss models is limited, the error characteristics are well known; these models enable the simple system design. However, because empirical models generalize radio propagation environments to typical environments such as urban, suburban, and rural, the propagation model cannot include site-specific dispersions: the estimation accuracy is limited [2]. In addition, although shadowing and fading are modeled by probability distribution such as log-normal distribution, Nakagami–Rice distribution and Rayleigh distribution, it is difficult to predict the deterministic propagation loss by a terminal alone. Furthermore, although there are several composite models considering shadowing and fading distributions, these models have high complexity and suitable model depends on the surrounding environment [3,4,5,6,7].

In order to solve the problem, a radio environment database based on measurement results has attracted attention in recent years [8,9,10]. The database first collects the radio propagation information from the mobile terminals. The datasets are then used to generate the statistical radio propagation information such as average received signal strength. Spectrum sharing over television white spaces (TVWSs) can be cited as an example of the application [11,12,13]. In the database-assisted spectrum sharing systems, we can estimate the white spaces by referring to the database. In addition, in the cellular systems, the database enables accurately estimating the communication coverage. However, the usage scenes of the conventional database are only limited to the communication systems that the transmitter is fixed in a given location [14,15,16,17,18,19,20,21]. Therefore, it is difficult to predict the site-specific propagation components such as shadowing in the wireless distributed network situations where both transmitter and receiver have arbitrary locations. On the other hand, even in such a distributed network, the site-specific components are uniquely determined according to the positional relationship between the transmitter and the receiver. Therefore, by relating the received signal power with the transmission/reception position and by reporting it to the external database, we will generate the average received power map for each transmission position. Reference [22] discusses a similar problem. The authors developed a distributed radio environment map cartography. In this method, distributed cognitive radio terminals preliminarily collect radio environment information by exchanging training symbols with each other. The channel gain in an arbitrary link is then tracked by utilizing Kriged Kalman filtering. However, this paper is a simulation-based study, and assumes that the receiver can remove the effect of multipath fading perfectly: the question remains in the feasibility.

In this paper, we propose a novel architecture of the measurement-based radio environment database for the wireless distributed network situations. In the proposed database, the datasets of different transmitters and different receivers are gathered by users obtaining from the usual communication packets. Such kind of data gathering enabled by distributed users is called *crowdsourcing*. By using crowdsourcing, the huge number of datasets can be gathered from huge number of locations. In order to evaluate the accuracy of the database, a measurement campaign with Vehicle-to-Vehicle (V2V) communication is conducted over a suburban area in Japan. In IEEE 802.11p, the existing communication standard for the V2V, the transmitter position is typically included into the transmission packet for traffic safety support. Therefore, by using the global positioning systems at the received vehicle, the Received Signal Strength Indicator (RSSI) and the both transmitter/receiver position can be stored without additional operation. After the observation experiment, we create radio environment maps (REMs) related to the transmission positions using MySQL server. Experimental results show that the RSSI can be accurately predicted compared with the path loss-based method. In addition, we apply the proposed database to the power control in the unicast system in order to discuss the effect of the proposed database on the communication efficiency. Evaluation results show that the proposed method can minimize the transmission power, i.e., interference with vehicles other than the destination, while guaranteeing the desired communication quality.

This paper is organized as follows. Section 2 shows the overview of the radio environment database for wireless distributed networks. Section 3 describes the measurement setup of the database construction. After we discuss the experimental results in Section 4, Section 5 concludes this paper.

Note that this paper is a revised and extend version of our work presented in Reference [23]. Based on this initial study, we newly discuss the proposed database-assisted power control in order to investigate the communication efficiency. In addition, in the discussion of the accuracy of the proposed method, a comparison of accuracy with other path loss models, the Okumura–Hata model and the two-ray path loss model is newly added.

## 2. Overview of Proposed Radio Environment Database

**Figure 1:**
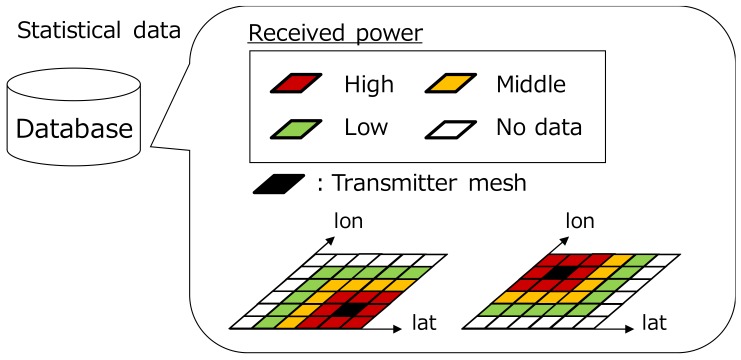
Concept of a radio environment database for wireless distributed networks.

Figure 1 shows an overview of the radio environment database for wireless distributed networks. The database splits the communication area into the two-dimensional meshes. By regarding each mesh as a transmission location, the database creates the REMs linked with each mesh: *N* maps are stored at the maximum when the area is divided into *N* meshes. Although there are several methods for the map construction such as empirical path loss models and ray tracing, we focus on the measurement-based approach. In this way, it becomes possible to grasp statistical radio environment characteristics based on measurement data and radio environment can be estimated more accurately than the existing propagation model.

**Figure 2:**
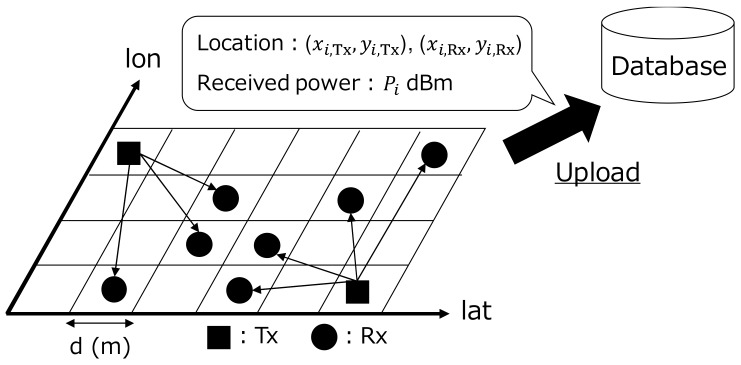
Data collection for database construction.

As shown in Figure 2, the transmitting vehicle firstly transmits a signal to the surrounding receiving vehicles. Here, we assume that the packet includes the transmission position and the ID of the transmitter. This is a reasonable assumption in the V2V systems because the packet in IEEE 802.11p usually includes these information for the traffic safety support. The receiver extracts these information, and stores with the reception position, the received signal power, the reception time, the operating frequency and the ID of the receiver. The stored information is reported to the database when the vehicle can be online with some methods such as cellular and Wi-Fi. In the practical situation, we should consider the effect of the upload procedure on the communication traffics. To offload the upload cost, it is better to upload the measurement data in the time of low communication traffic, such as uploading via home Wi-Fi at night. After the database collects massive communication results, we create the REMs by utilizing the datasets. When multiple reported value data are included in a mesh, the mesh calculates the average value of the received power.

The dataset accumulated after the initial database construction should be utilized again for averaging. This is because the average characteristic of radio propagation, i.e., sum of path loss and shadowing, is generally static over time domain. We anticipate that the accuracy of averaged multipath fading improves, as the number of samples increases. On the other hand, in the realistic situation, the shadowing effect will fluctuate when there is a change in the environment of the structure. One candidate of the countermeasure for this problem is to investigate the change of structure by time series analysis of observed values such as comparing the latest observation result with the probability density function (PDF) that consists of past observation results. If a change of the structure is detected, we can reconstruct the accurate database by removing the past observation results for the database construction or by taking weighted averaging with forgetting factors. Because this problem requires new algorithms and is far beyond the focus of this paper, we consider that this is a future work.

After the database construction, the transmitter can adjust the communication parameters by utilizing the statistical information stored in the database. When the transmitter accesses to the database, the database provides the map corresponding to the transmitter position. By using the map, the transmitting vehicle can confirm the statistical communication quality at the arbitrary received location, and can modify the communication parameters. For example, when the estimated received signal power is lower than the desired value, the transmitter can improve the packet reception probability by using higher transmission power, low rate modulation schemes, and relay communications. In addition, if the estimated quality is higher than the desired value, the transmitter can choose the high rate communication with higher-order modulation or the interference mitigation with lower transmission power. The effect of the database-assisted communication parameter setting is discussed in Section 4.4.

Note that this paper does not consider the use of spatial interpolation for the REM construction. This is because the main purpose of this paper is to show that the proposed database can estimate the site-specific propagation attenuation using meshes related to the pair of transmission and reception locations. It should be noted that the spatial interpolation can be implemented by the path loss model that is fitted to the actually observed datasets. This is discussed in Section 4.

## 3. Measurement Campaign

In order to construct a radio environment database for wireless distributed networks and evaluate the accuracy of the database, V2V communications were conducted in Chofu City and Mitaka City, typical suburban areas in Tokyo, Japan over three days in January 2017. Measurement conditions are shown in Table 1. Note that the measurement setup, procedure, and the database structure are the same as in our initial work [23].

### 3.1. Measurement Equipment

**Figure 3:**
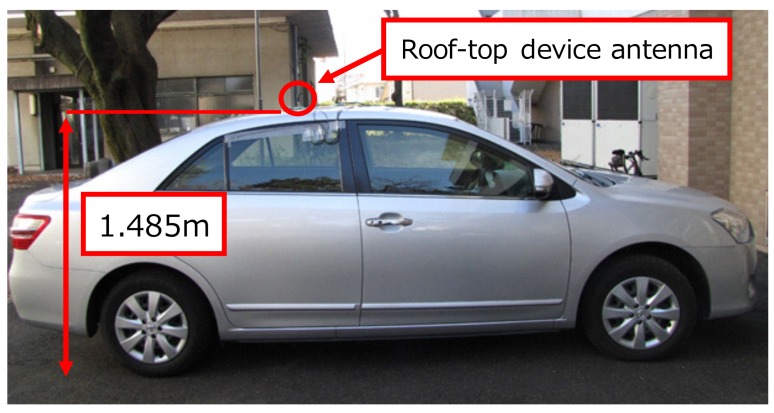
Observation vehicle.

**Figure 4:**
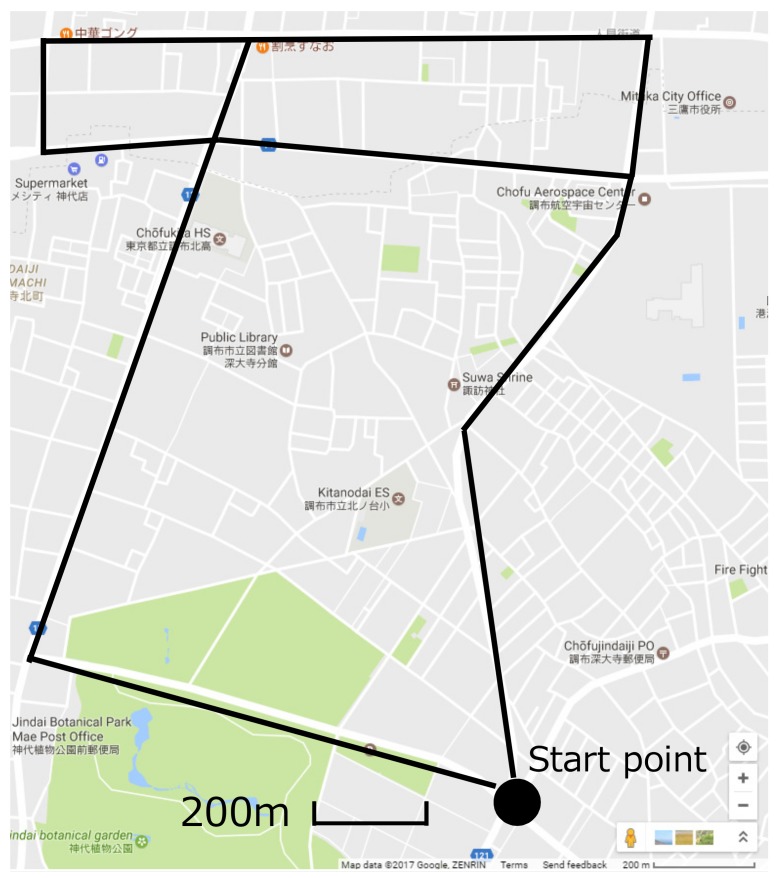
Measurement route.

Three vehicles shown in Figure 3 were prepared, and each vehicle was implemented in an in-vehicle device. For gathering the radio environment information, the vehicles communicated each other while traveling on the route shown in Figure 4. The speed of the vehicle is 40 km/h because this is the legal speed of vehicles in the metropolitan area in Japan. ARIB STD-T109 standard transmitters (Denso Corporation, Kariya, Japan) and receivers are used for V2V communication. This standard is Japanese V2V communication based on IEEE 802.11p and operated over 760 MHz. The modulation format is Orthogonal Frequency Division Multiplexing (OFDM), the access format is Carrier Sense Multiple Access/Collision Avoidance (CSMA/CA), and the protocol stack is IEEE 802.11p based physical layer.

A signal is transmitted from the onboard device at a cycle of 100 ms, and the other vehicle records the received signal power, the received time, the ID of both transmitter and receiver, and the received/transmitted position. Note that the transmitted position was extracted from the transmitted packet, and the received position and the time was obtained from the GPS connected to the onboard device. Note that Garmin GPS 18x, a USB-connected device, was used for the GPS system. The GPS acquires the location and time information once per second, and the accuracy of the position information is 95% within 15 m.

Here, specifications of the onboard device are shown in Table 2. The transmission time of one packet is 232 μs, the transmission cycle is 100 ms, and the position information updating period is 200 ms. Therefore, the vehicle device updates the position information every two messages. In addition, monopole antennas are used for both the reception and transmission. Table 3 shows the antenna characteristics.

### 3.2. Statistical Processing with Radio Environment Database

**Figure 5:**
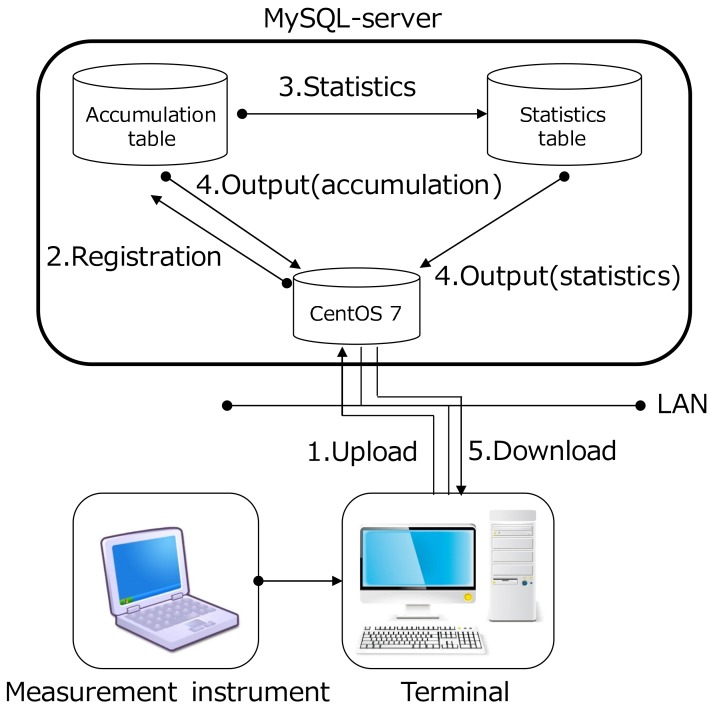
Structure of the implemented database.

A radio environment database was constructed by statistical process to the measured datasets. Figure 5 shows the overview of the database. The database is operated by Cent OS 7 and MySQL 5.7, and the statistical processing is implemented by Hypertext Preprocessor (PHP) programs. After the measurement campaign, the statistical processing is performed as follows:

1. The measured datasets are reported from each vehicle to the database.
2. The database server registers the reported datasets to the table in the MySQL server.
3. The database server performs the statistical processing of the dataset. In this scheme, the database calculates the average RSSI for each pair of transmission and reception meshes using a PHP program. Then, the calculated statistical data is stored in the table for the statistical data.
4. According to the request from the terminal, the database server attempts to get the statistical data from the MySQL.
5. The terminal can get the radio environment maps.

**Figure 6:**
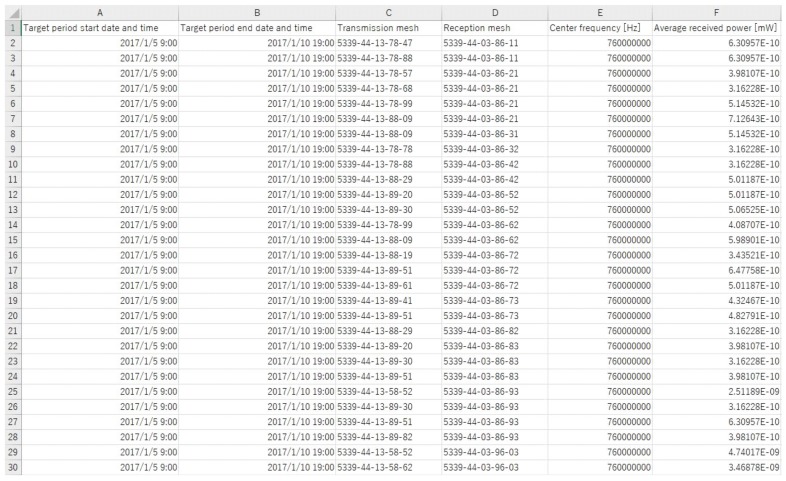
Example of the statistical data.

In our measurement campaign, the receiving vehicles record the above information into a comma-separated values (CSV) file when the packet is successfully decoded. The stored CSV file is uploaded to the database server offline after the measurement campaign. In the database server, the RSSI is stored for each pair of transmission and reception meshes and statistical processing is performed. Here, transmission and reception meshes are calculated by normalizing latitude and longitude in the database server. In the local PC, download the statistical data in which RSSI is averaged for each pair of transmission and reception meshes in the CSV file. Figure 6 shows the example of the statistical data. Then, we specify any transmission mesh and create the radio environment map by using Python script. Finally, we can download the CSV file and create a map with Python script. If the new datasets are observed after the initial creation of the map, the new datasets are added to the accumulation table in the database server and calculate the average RSSI for each pair of transmission and reception meshes again. Table 4 and Table 5 show registration data and statistical data in the MySQL server, respectively. The mesh code (First, Second, Third…) shows the scale of communication area and the smaller the mesh code, the more detailed radio environment can be grasped. The mesh code is calculated from latitude and longitude and used to conduct REMs. Transmitter ID and Receiver ID are used to delete unnecessary datasets. Statistical data represents the radio environment in the communication area and REMs are conducted by using Transmitter mesh code, Receiver mesh code and Averaged received power in dBm for each Transmitter mesh.

## 4. Experimental Results

In this section, we discuss the constructed database. After several maps are shown as examples, the accuracy of the database is evaluated by using Root Mean Squared Error (RMSE).

### 4.1. Example of REMs

**Figure 7:**
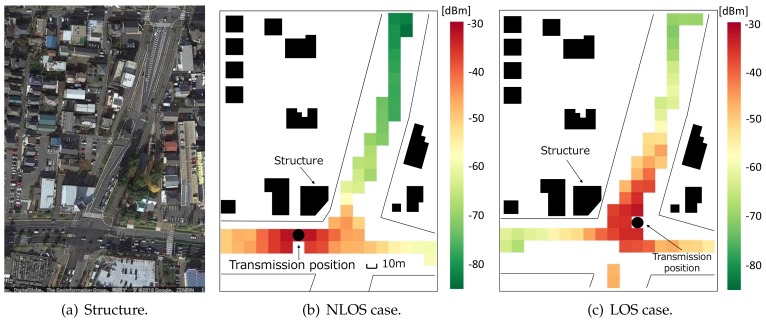
Examples of radio environment maps.

Figure 7b,c show examples of the constructed maps in Figure 7a. In these figures, the datasets were processed with a 10 m-squared mesh. Each map has different transmission positions, and we can confirm the effect of obstacles on the radio environment characteristics. As can be seen from the maps, the received signal power characteristics obviously fluctuate by changing the reference transmission mesh. For example, the difference of around 20 dB can be confirmed between two maps on the north side of the area. In Figure 7b, since the transmission position is located in front of the structure, the communication between the transmitter and the north area becomes a non-line-of-sight (NLOS) environment. On the other hand, line-of-sight (LOS) communications can be conducted in Figure 7c: the obstacle-dependent signal attenuation does not occur in this case.

These facts suggest that the measurement-based maps can contain the effect of structure in the radio propagation estimation. The accuracy of the constructed database is evaluated in Section 4.3. Here, because only the route where the vehicles traveled has the average RSSIs, the map has tooth-missing information. On the other hand, in actual operation, massive numbers from the communication log will be obtained from many crowdsourcing vehicles. This means that there will be no data loss on the road where we usually drive.

In addition, we evaluated the calculation time for statistical processing of the datasets. Statistical processing is implemented by PHP 7.0.12 in Cent OS 7 with Intel(R) Xeon(R) CPU E5-2407@ 2.20 GHz. Table 6 summarizes examples of the calculation times for the statistical processing. The first row of the Table 6 is all datasets in this measurement campaign.

### 4.2. Propagation Characteristics

We derive the shadowing characteristics in this experimental environment to indicate that the characteristics show a good match with the general suburban area. First, the statistical processing with 10 m-squared meshes is performed by using all datasets. To derive the path loss characteristic, the link distances in the datasets were obtained from the transmitter/receiver position information. The logarithm of the link distance *d* (m) is then calculated and the scatter diagram was obtained. Note that the noise floor of our equipment in the communication bandwidth is about −96.0 dBm. In order to eliminate effects of the noise floor, we limit the maximum distance in the performance evaluation between links to 100 m. If we process the datasets over longer communication distances, the average received power is overestimated because we cannot obtain RSSIs below the noise floor. Because this is a challenging problem, this paper saves this issue for a future work. A similar problem is pointed out in some papers (e.g., Reference [24]). Referring to such a discussion, the overestimation will be improved.

In this paper, we model the distance attenuation characteristics as

(1) $$\begin{array}{c} PL\left(d\right)=\left\{\begin{array}{cc} a_{1}\log_{10}\left(d\right)+b_{1} & \left(d\leq R_{b}\right)\\ a_{2}\log_{10}\left(d\right)+b_{2} & \left(d>R_{b}\right) \end{array}\right.\;\;\;\left(\mathrm{dB}\right), \end{array}$$

where $a_{1},a_{2}$ are the path loss index factor of the location dependency and $b_{1},b_{2}$ are the constant value that contains transmission power and antenna effects. $R_{b}$ is the distance from transmission position to break point and is expressed as follows [25]:

(2) $$R_{b}=\frac{4h_{b}h_{m}}{\lambda }\;\;\;\left(m\right),$$

where $h_{b}$ and $h_{m}$ are the transmitter and receiver height including antenna height, and these values are 1.485 m, respectively. $a_{1},a_{2}$ and $b_{1},b_{2}$ were obtained from the scatter diagram by using the least squares method, and the path loss characteristic was estimated. From the least squares method, we obtained the distance attenuation characteristic in this communication area as follows:

(3) $$\begin{array}{c} PL\left(d\right)=\left\{\begin{array}{cc} -5.4919\log_{10}\left(d\right)-30.1360 & \left(d\leq R_{b}\right)\\ -31.925\log_{10}\left(d\right)+7.9852 & \left(d>R_{b}\right) \end{array}\right.\;\;\;\left(\mathrm{dB}\right). \end{array}$$

**Figure 8:**
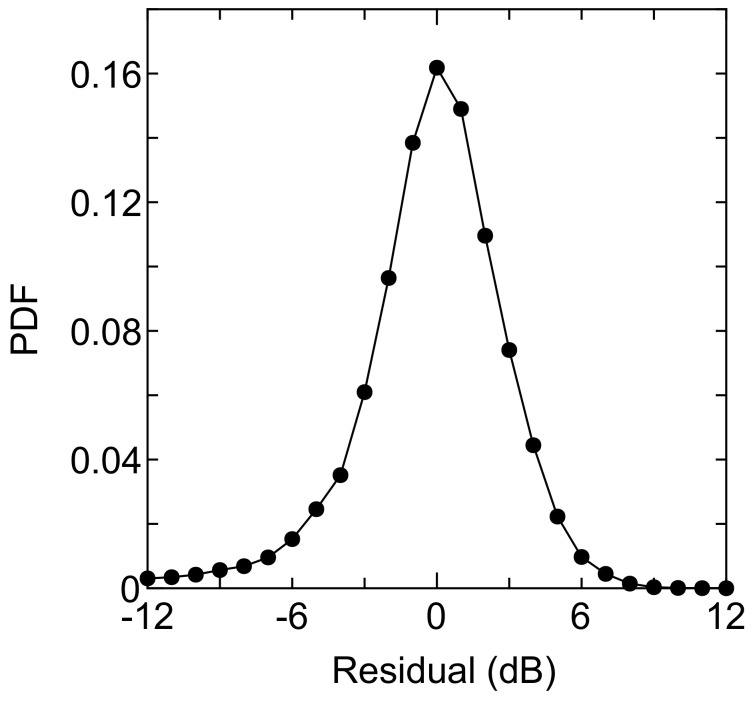
Probability density function of the shadowing component.

Therefore, the path loss index can be calculated as 3.1925 for $d>R_{b}$. The shadowing components were then obtained by subtracting the estimated path loss from the average received power in each mesh. As shown in Figure 8, the lognormal-like distribution can be confirmed. Here, the logarithmic mean is μ=0.0005198 dB, and the standard deviation is σ=3.776 dB. These values match well with the empirical values in typical suburban areas [26,27,28].

### 4.3. Estimation Accuracy

Next, we evaluate the accuracy of the constructed database. We constructed the database using the datasets observed on days 1 and 2. Statistical processing was performed with 2 m-, 5 m-, and 10 m-squared meshes, and the average received power value for each mesh was calculated. The datasets observed on day 3 were treated as the instantaneous signal power, and RMSE was calculated from the difference between the constructed database and the instantaneous values.

**Figure 9:**
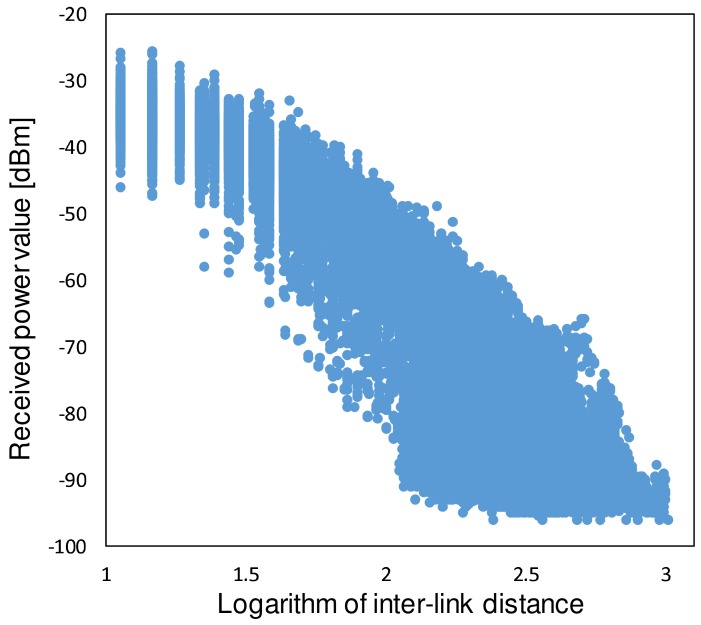
Average received power value and distance attenuation characteristics.

For comparison, path loss-based estimation method is also evaluated. The model in this communication area was calculated from the datasets observed on days 1 and 2. Although there are more complex path loss models that consider a larger number of variables (i.e., terrain models), they do not necessarily make better predictions [2]. Thus, the fitted path loss is a simple and accurate candidate for the compared path loss model. The scatter diagram is shown in Figure 9. The path loss was derived by considering the break point. From the figure, the path loss with the datasets was estimated as

(4) $$\begin{array}{c} PL\left(d\right)=\left\{\begin{array}{cc} -8.5478\log_{10}\left(d\right)-25.795 & \left(d\leq R_{b}\right)\\ -43.364\log_{10}\left(d\right)+27.312 & \left(d>R_{b}\right) \end{array}\right.\;\;\;\left(\mathrm{dB}\right). \end{array}$$

Note that parameters in this equation are different from Equation (3) because Equation (3) is estimated by using datasets observed on all the measurement days. On the other hand, Equation (4) consists of datasets obtained only on days 1 and 2.

In the path loss-based method, the estimated received power is derived by subtracting the propagation loss corresponding to Equation (4) from the transmission power. Then, we calculate the RMSE by taking the difference between the estimated received power and the datasets observed on day 3.

In addition, we compare the estimation accuracy to the ITU-R P.1411 [25]. In this evaluation, we calculated attenuation based on ITU-R P.1411 by using the following equation:

(5) $$\begin{array}{c} L_{m}\left(d\right)=L_{b}+6+\left\{\begin{array}{cc} 20\log_{10}\left(\frac{d}{R_{b}}\right) & \left(d\leq R_{b}\right)\\ 40\log_{10}\left(\frac{d}{R_{b}}\right) & \left(d>R_{b}\right) \end{array}\right.\;\;\;\left[\mathrm{dB}\right], \end{array}$$

where *d* [m] is inter-link distance. $L_{b}$ is propagation loss from transmitter position to break point and $R_{b}$ is the distance from transmission position to break point. They are expressed as follows:

(6) $$L_{b}=\left|20\log_{10}\left(\frac{\lambda^{2}}{8\pi h_{b}h_{m}}\right)\right|\;\;\;\left[\mathrm{dB}\right],$$

(7) $$R_{b}=\frac{4h_{b}h_{m}}{\lambda }\;\;\;\left[m\right].$$

Estimated received power was derived by subtracting the propagation loss that corresponded to Equation (5) from transmission power and calculated RMSE from the difference between the estimated received power and the day 3 datasets.

In addition, we compare the estimation accuracies of the two-ray path loss model [29] and the Okumura–Hata model [30].

**Figure 10:**
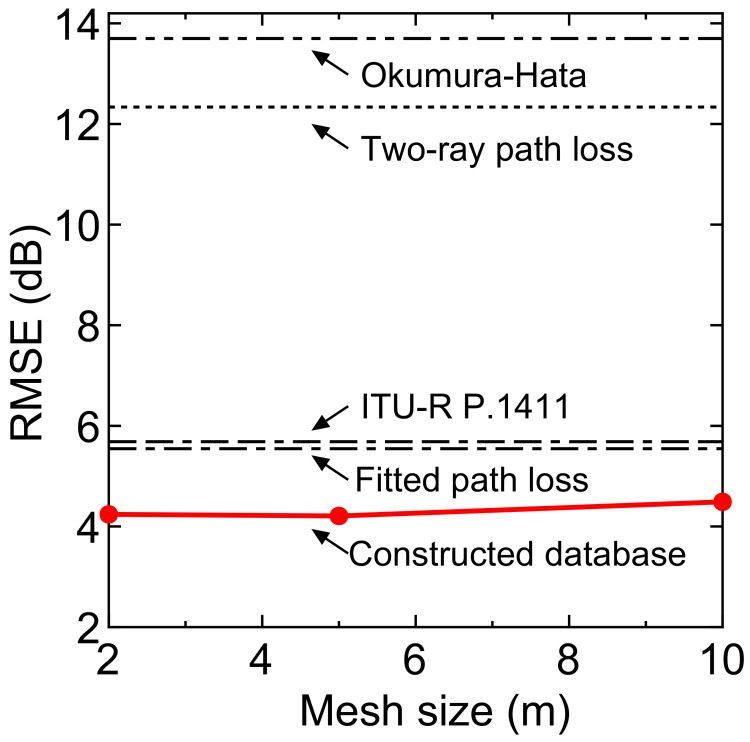
Mesh size versus RMSE.

Figure 10 shows the RMSE characteristics. The RMSE of the fitted path loss model was 5.546 dB. On the other hand, the RMSE of the constructed database are 4.241 dB at 2 m-squared mesh, 4.210 dB at 5 m-squared mesh, and 4.489 dB at 10 m-squared mesh. The reason why the RMSE at the 2 m-squared mesh is slightly large is that the number of data hits in the search was reduced due to the reduction of the mesh size, and a sufficient amount of data could not be obtained. Considering the standard deviation of the shadowing component and the instantaneous fading component, we can consider that the RMSE derived by this accuracy evaluation is generally a good value. The RMSE of the two-ray path loss model, the Okumura–Hata model, and ITU-R P.1411 were 12.34 dB, 13.70 dB and 5.67 dB, respectively. From the above results, we can conclude that more detailed signal fluctuation can be predicted by using the measurement-based radio environment database in the V2V communication systems. The proposed database will enable the improvement of the communication efficiency in the V2V communications.

Here, it should be noted that the fitted path loss can be used for estimating the received signal power where there is no statistical data. Thus, the RMSE of the fitted path loss also shows the performance of fitted path loss-aided interpolation.

### 4.4. Power Control Based on the Proposed Database

Finally, we discuss the effect of the proposed database-assisted communications. In the unicast communication, unlike the broadcast communication, the transmitted signal becomes the interference power at the vehicles other than the destination. Thus, suppressing the transmission power to the minimum, i.e., suppressing the spatial interference power, improves the communication efficiency. Motivated by this fact, we consider a simple power control situation where the transmitter designs its own transmission power according to the database so that we guarantee the outage probability that the received signal power falls below the desired value. The performance of the power control is performed according to the following procedure:

1. The database with 10-m mesh is constructed with the datasets observed on days 1 and day 2.
2. The empirical cumulative distribution function (CDF) of multipath fading factor in the evaluated area is estimated by taking the difference between each instantaneous dataset used for the database construction and the average received signal power in the database. We assume that the shape of CDF is static in the evaluation area.
3. The datasets observed on day 3 are treated as the instantaneous true received signal power. The database estimates the instantaneous received power at the reception mesh via the constructed database.
4. The database calculates the transmission power from the estimated average received signal power and the empirical CDF of the multipath fading so that the outage probability is guaranteed. Note that we limit the maximum transmission power to 19.2 dBm, which is the rated output in our equipment.
5. This power control is applied for all the instantaneous datasets observed on day 3.

The evaluation is performed around the intersection shown in Figure 7. For the performance comparison, we also perform the empirical path loss-based power control with the same procedure. In this method, the average received power is estimated via the measurement-based path loss model fitted to Equation (1). By fitting the model with the datasets from day 1 and day 2, we estimated the path loss as

(8) $$\begin{array}{c} PL\left(d\right)=\left\{\begin{array}{cc} -9.6183\log_{10}\left(d\right)-24.335 & \left(d\leq R_{b}\right)\\ -43.470\log_{10}\left(d\right)+26.699 & \left(d>R_{b}\right) \end{array}\right.\;\;\;\left[\mathrm{dB}\right]. \end{array}$$

Note that parameters in this equation are different from Equations (3) and (4) because Equation (8) is estimated with datasets obtained in the intersection shown in Figure 7. On the other hand, both Equations (3) and (4) are estimated by the datasets obtained over the entire measurement area.

**Figure 11:**
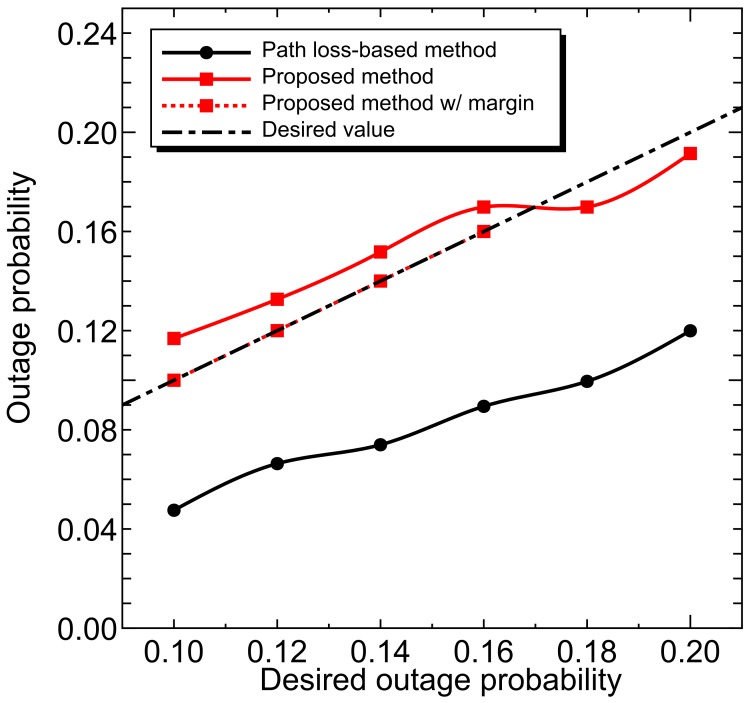
Outage probability characteristics after power control.

We performed the power control where the permissible outage probability is 0.1–0.2 and the desired received power is –82.0 dBm. Figure 11 shows the outage probability. Because the path loss-based estimation contains the uncertainties of both shadowing and multipath fading effects, the method overestimates the transmission power, and the outage probability is much lower than the desired value. On the other hand, the performance of the proposed method nearly equals the desired value. Here, we can confirm that the outage probability in the proposed method slightly exceeds the setting value when the value is small. This is due to the following reasons:

- Uncertainty of estimation of the tail in the empirical CDF. Although achieving a high packet delivery ratio requires accurate information of the tail, it is difficult to obtain such information in the empirical CDF with limited samples.
- Limitation of the maximum transmission power.

**Figure 12:**
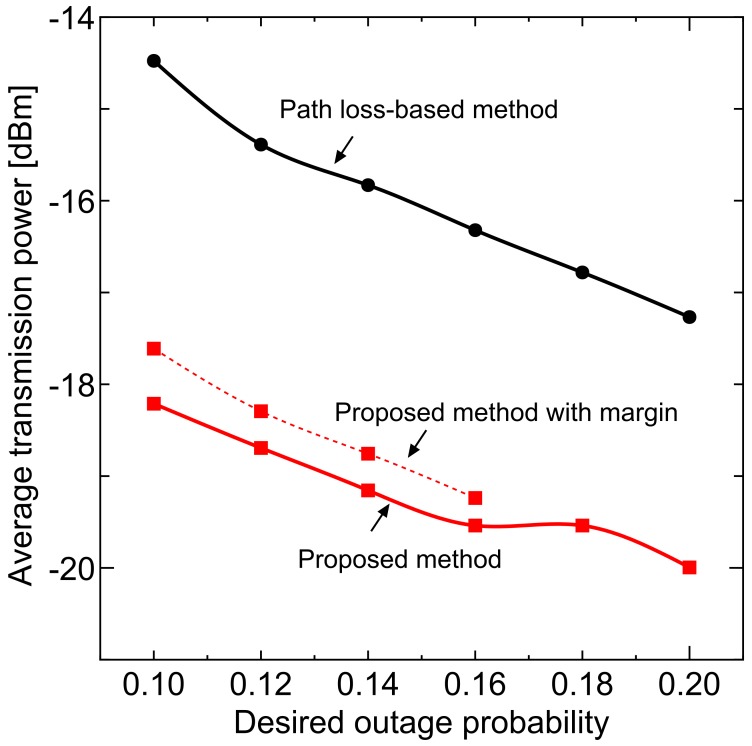
Average transmission power.

In order to keep the permissible outage probability under the above conditions, we set the margin to the transmit power. The dotted line in Figure 11 shows the outage probability with margin and Figure 12 shows the average transmission power including setting margin. In addition, for the region where the proposed method does not satisfy the desired value, the margin that satisfies the desired value is separately evaluated and its performance is plotted with the dotted line.

The proposed method can achieve the desired value with a small margin of less than 1 dB and, even if the margin is taken into consideration, the average transmission power is lower than the path loss based method. These results mean that the proposed method can achieve the desired communication quality with lower transmission power: spectral efficiency can be improved.

## 5. Conclusions

The measurement-based radio environment databases were proposed in the V2V communication environment. The evaluation results showed that the proposed database accurately estimates the radio environment fluctuations compared to some path loss models including the measurement-based path loss model and ITU-R P.1411. We conclude that the measurement-based radio environment database can be used for predicting radio environment characteristics in not only conventional applications where the transmitter is fixed, but also V2V communication systems.

In addition, we have shown that the proposed database can improve power control in the unicast situation, and the proposed database will also improve the communication efficiency in V2V systems.

## Figures and Tables

**Table 1:** Measurement conditions.

Observation time	9:00 a.m. to 6:00 p.m.
Line of Sight (LOS) environment	straight road
Non Line of Sight (NLOS) environment	intersections

**Table 2:** Specifications of on-vehicle devices.

Communication standard	ARIB STD-T109
Modulation method	OFDM/QPSK(1/2)
Transmission power (mW)	83
Center frequency (MHz)	760
Current consumption (A)	0.5
Communication header (byte)	61
Payload length (byte)	77

QPSK: Quadrature Phase Shift Keying).

**Table 3:** Antenna characteristics.

Item	Specification
Type	Monopole antenna
Frequency range	755–765 MHz
Absolute gain	2.15 dBi
Element length	110 mm

**Table 4:** Registration data.

Item	Type	Size (Byte)	Remarks
Measurement datetime	datetime	8	YYYY/MM/DD hh:mm:ss
Transmission latitude	double	8	Transmission latitude (${}^{\circ }$)
Transmission longitude	double	8	Transmission longitude (${}^{\circ }$)
Reception latitude	double	8	Reception latitude (${}^{\circ }$)
Reception longitude	double	8	Reception longitude (${}^{\circ }$)
Altitude	double	8	Altitude (m)
Center frequency	double	8	Center frequency (Hz)
Received Signal Strength Indicator (RSSI)	double	8	Received Signal Strength Indicator (mW)
Packet ID	integer	4	-
Transmitter ID	char	17	-
Receiver ID	char	17	-
Transmitter mesh code (First)	char	4	XXXX
Transmitter mesh code (Second)	char	2	XXXX-XX
Transmitter mesh code (Third)	char	2	XXXX-XX-XX
Transmitter mesh code (1/10)	char	2	XXXX-XX-XX-XX
Transmitter mesh code (10 m)	char	2	XXXX-XX-XX-XX-XX
Transmitter mesh code (5 m)	char	3	XXXX-XX-XX-XX-XX-XX
Transmitter mesh code (2 m)	char	3	XXXX-XX-XX-XX-XX-XX
Transmitter mesh code (1 m)	char	3	XXXX-XX-XX-XX-XX-XX
Receiver mesh code (First)	char	4	XXXX
Receiver mesh code (Second)	char	2	YYYY-YY
Receiver mesh code (Third)	char	2	YYYY-YY-YY
Receiver mesh code (1/10)	char	2	YYYY-YY-YY-YY
Receiver mesh code (10 m)	char	2	YYYY-YY-YY-YY-YY
Receiver mesh code (5 m)	char	3	YYYY-YY-YY-YY-YY-YY
Receiver mesh code (2 m)	char	3	YYYY-YY-YY-YY-YY-YY
Receiver mesh code (1 m)	char	3	YYYY-YY-YY-YY-YY-YY
Saved date	datetime	8	Data registration date and time

**Table 5:** Statistical data.

Item	Type	Size (Byte)	Remarks
Target period (start)	datetime	8	YYYY/MM/DD hh:mm:ss
Target period (end)	datetime	8	YYYY/MM/DD hh:mm:ss
Transmitter mesh code	char	20	specified transmitter mesh code
Receiver mesh code	char	20	specified receiver mesh code
Transmitter southwest latitude	double	8	Transmitter southwest latitude (${}^{\circ }$)
Transmitter southwest longitude	double	8	Transmitter southwest longitude (${}^{\circ }$)
Transmitter northeast latitude	double	8	Transmitter northeast latitude (${}^{\circ }$)
Transmitter northeast longitude	double	8	Transmitter northeast longitude (${}^{\circ }$)
Receiver southwest latitude	double	8	Receiver southwest latitude (${}^{\circ }$)
Receiver southwest longitude	double	8	Receiver southwest longitude (${}^{\circ }$)
Receiver northeast latitude	double	8	Receiver northeast latitude (${}^{\circ }$)
Receiver northeast longitude	double	8	Receiver northeast longitude (${}^{\circ }$)
Averaged received power	double	8	Averaged received power (mW)
Saved date	datetime	8	Data registration date and time

**Table 6:** Calculation time for statistical processing.

Day	Number of Dataset	Number of Registered Mesh	Calculation Time (s)	Average Registration Time per Mesh (s)
All days	2839101	69158	24.0	0.00036
Days 1 and 2	1940364	53096	17.0	0.00033
Day 3	898691	37786	10.0	0.00028
